# Oral and long-acting injectable antipsychotic discontinuation and relationship to side effects in people with first episode psychosis: a longitudinal analysis of electronic health record data

**DOI:** 10.1177/20451253231211575

**Published:** 2023-12-15

**Authors:** Rashmi Patel, Aimee Brinn, Jessica Irving, Jaya Chaturvedi, Shanmukha Gudiseva, Christoph U. Correll, Paolo Fusar-Poli, Philip McGuire

**Affiliations:** Department of Psychological Medicine, Institute of Psychiatry, Psychology and Neuroscience, King’s College London, De Crespigny Park, Denmark Hill, London, SE5 8AF, UK; Department of Psychological Medicine, Institute of Psychiatry, Psychology and Neuroscience, King’s College London, London, UK; Department of Psychological Medicine, Institute of Psychiatry, Psychology and Neuroscience, King’s College London, London, UK; Department of Psychological Medicine, Institute of Psychiatry, Psychology and Neuroscience, King’s College London, London, UK; South London and Maudsley NHS Foundation Trust, London, UK; Department of Child and Adolescent Psychiatry, Psychosomatic Medicine and Psychotherapy, Charité – Universitaetsmedizin Berlin, corporate member of Freie Universitaet Berlin, Humboldt Universitaet zu Berlin, and Berlin Institute of Health, Berlin, Germany; Department of Psychiatry, The Zucker Hillside Hospital, Northwell Health, Glen Oaks, NY, USA; Department of Psychiatry and Molecular Medicine, Zucker School of Medicine at Hofstra/Northwell, Hempstead, NY, USA; South London and Maudsley NHS Foundation Trust, London, UK; Department of Psychosis Studies, Institute of Psychiatry, Psychology and Neuroscience, King’s College London, London, UK; Department of Brain and Behavioural Sciences, University of Pavia, Pavia, Italy; Department of Psychiatry, University of Oxford, Oxford, UK; Oxford NIHR Biomedical Centre, Oxford, UK

**Keywords:** Real-World Data, RWD, FEP, EHR, epidemiology, EPSE, hyperprolactinemia, sedation, sexual, weight gain

## Abstract

**Background:**

Discontinuation of treatment in people with first episode psychosis (FEP) is common, but the extent to which this is related to specific adverse effects of antipsychotic medications is unclear.

**Objectives:**

To investigate whether antipsychotic discontinuation is associated with the prescription of particular antipsychotics and particular adverse effects.

**Design:**

Retrospective cohort study.

**Methods:**

We assembled de-identified electronic health record (EHR) data from 2309 adults with FEP who received care from the South London and Maudsley NHS Foundation Trust between 1st April 2008 and 31st March 2019. Associations between antipsychotic medications, clinician-recorded side effects and treatment discontinuation were investigated across a mean follow-up period of 34.2 months using Cox regression.

**Results:**

The mean age of patients was 26.7 years and 1492 (64.6%) were male. Among first prescribed antipsychotic medications, discontinuation occurred earlier with haloperidol [hazard ratio (HR) = 2.78, 95% CI = 1.69–4.60] and quetiapine (HR = 1.43, 95% CI = 1.16–1.80) than with olanzapine. Discontinuation occurred sooner when there was evidence of extrapyramidal symptoms (HR = 1.33, 95% CI = 1.08–1.64) or sexual dysfunction (HR = 1.59, 95% CI = 1.03–2.46). Among antipsychotics prescribed at any point during treatment, lurasidone (HR = 1.40, 95% CI = 1.10–1.78) and aripiprazole (HR = 1.09, 95% CI = 1.01–1.19) were associated with earlier discontinuation than olanzapine. Conversely, clozapine (HR = 0.55, 95% CI = 0.41–0.73) and paliperidone 1-monthly (PP1M) long-acting injectable (HR = 0.80, 95% CI = 0.68–0.94) were associated with later discontinuation. Unexpectedly, for antipsychotics prescribed at any stage of treatment, sedation (HR = 0.89, 95% CI = 0.81–0.97), weight gain (HR = 0.73, 95% CI = 0.64–0.83), and multiple side effects (HR = 0.83, 95% CI = 0.76–0.90) were associated with later discontinuation.

**Conclusion:**

Earlier treatment discontinuation associated with sexual or extrapyramidal side effects could be related to their rapid onset and poor tolerability. Later treatment discontinuation associated with clozapine and PP1M could be related to the relative efficacy of these treatments. These findings merit consideration when selecting antipsychotic therapy for people with FEP.

## Introduction

Antipsychotic medications are the first-line treatment for individuals with a first episode of psychosis (FEP).^1^ These drugs are broadly similar in efficacy and their mechanism of action, but vary significantly in their side effect profile.^2–4^ International guidelines do not recommend a particular medication for the first antipsychotic treatment.^5^ Rather, clinicians are encouraged to tailor treatment choice for each individual according to side effect profile.^1,2,6^

Most people with FEP show a symptomatic improvement following their first course of treatment with an antipsychotic medication.^3,7^ However, many of these patients subsequently discontinue treatment,^8,9^ with discontinuation rates of 73–77% within 6–12 months.^10,11^ This may be due to a lack of efficacy. However, paradoxically, it can also result from patients being more sensitive to the therapeutic effects of antipsychotic medications at this stage,^12^ with the relatively rapid resolution of symptoms leading to patients feeling that medication is no longer needed.^13^ People with FEP are also more sensitive to the adverse effects of antipsychotics than chronic patients.^14^ Interruption of antipsychotic treatment is associated with a five-fold increase in the risk of subsequent relapse.^15^

In people with chronic schizophrenia, time to discontinuation is significantly longer for those taking olanzapine than those taking quetiapine or risperidone.^16,17^ Less is known about whether there are differences in the discontinuation rates of antipsychotics being taken by people with FEP. A greater understanding of treatment discontinuation at this stage would inform the clinical management of medication nonadherence and efforts to reduce the risk of subsequent relapse.^12,18^,

In general, side effects are linked to reduced treatment adherence to medication in patients with FEP and chronic schizophrenia.^13,19^ However, the extent to which poor adherence is associated with particular adverse effects is unclear. Similarly, although medication discontinuation has been linked to Parkinsonian side effects,^20,21^ there is a lack of data on whether this also applies to other side effects.^3^

These issues can be addressed through large-scale studies of real-world clinical data. In the present study, we analysed electronic health records (EHRs) to investigate the prevalence of clinician-recorded antipsychotic side effects and their associations with treatment discontinuation in a large sample of patients with FEP. We hypothesized that clinician-recorded side effects are associated with a reduction in time to discontinuation of the first prescribed antipsychotic.

## Methods

### Aims

To assess:

(i) Time to discontinuation of different antipsychotics used as the first prescribed treatment after the diagnosis of FEP.
(ii) Time to discontinuation of different antipsychotics prescribed at any point during treatment.
(iii) Associations between clinician-recorded side effects and time to discontinuation of the first prescribed antipsychotic.
(iv) Associations between clinician-recorded side effects and time to discontinuation of antipsychotics prescribed at any point during treatment.

### Study setting

Data were extracted from the South London and Maudsley (SLaM) NHS Trust Biomedical Research Centre (BRC) case register. SLaM is a large UK health provider, which provides specialist mental healthcare to an area of 1.3 million residents four South London boroughs: Croydon, Southwark, Lewisham, and Lambeth.^22^ The clinical records of patients who received care from SLaM have been fully electronic since 2007.^22^ SLaM provides specialist Early Intervention Services (EIS) for patients with FEP and aims to increase early detection of psychosis, ensure timely treatment and improve access to psychological and pharmacological treatments for people experiencing their first episode of psychosis.

### Source of clinical data

The SLaM BRC Case Register contains de-identified, clinician-recorded EHR data for patients receiving care from SLaM clinical services. Data are entered by clinicians in structured fields (including demographics, psychotropic medications and contacts with mental healthcare services) and in unstructured ‘free text’ fields where narrative data are recorded, which describe the clinical presentation, progress and agreed treatment plan following contact between a patient and a clinician. The Clinical Interactive Record Search (CRIS) tool was used to extract data for the study. CRIS enables the query and assembly of de-identified EHR data in the SLaM BRC Case Register to support large-scale epidemiological analyses. The SLaM BRC Case Register and CRIS tool received ethical approval for use as a source of data for mental health research studies by the Oxfordshire Research Ethics Committee C (reference 08/H0606/71+5).

### Participants and inclusion criteria

Data were extracted for all individuals aged 16–65 years who were accepted into a SLaM EIS between 1st April 2008 and 31st March 2019. This time period was chosen to maximize the sample size of patients with at least 2 years of follow-up data. Patients aged between 16–65 years were included, as this is the intake age range for SLaM EIS.^23^ Follow-up data were obtained up to 31st March 2021. Patients were excluded if they did not have evidence of a documented start date of a prescribed antipsychotic medication. The index date was defined as the date of accepted referral to the SLaM EIS. Participants with missing gender or ethnicity data were excluded from the study.

### Data extraction

Data were extracted using CRIS tool through Structured Query Language queries. The following variables were extracted from structured data: age, gender, ethnicity, diagnosis, EIS team, index date and date of death (for patients who died during the study period). Ethnicity data were categorized according to the UK Office for National Statistics guidelines and recoded into four groups: ‘Asian’, ‘Black’, ‘Mixed’, ‘White’ and ‘Other’. The following variables were manually extracted from unstructured data: specific antipsychotics prescribed, antipsychotic start and stop date (if stopped during the study period), and clinician-recorded side effects [extrapyramidal side effect (EPSE), hyperprolactinemia, sedation, sexual, weight gain]. These side effects were chosen based on their potential associations with antipsychotics as documented in the Maudsley Prescribing Guidelines.^2^ For details of all structured and unstructured data variables, see Supplemental Tables 1 and 2. Supplemental Table 3 lists the names of antipsychotics ascertained from unstructured data. The list includes the generic and brand names of antipsychotics licensed for use in the United Kingdom during the study period.

### Unstructured data ascertainment

Unstructured data were defined as ‘free text’ documents (event notes, discharge summaries and clinical correspondence) recorded by clinicians in the SLaM BRC Case Register. Unstructured data for the patients included in the study were manually reviewed by two trained mental health researchers (AB and JI) to identify the variables described previously. Inter-rater reliability (IRR) figures for the ascertainment of unstructured data were estimated using a sample of 100 patient records which were reviewed by both AB and JI. IRR was high for antipsychotic name (Cohen’s kappa = 0.92) and start and stop dates (Cohen’s kappa = 0.89). Disagreements and questions during data ascertainment were resolved by a trained psychiatrist (RP).

### Data assembly and analysis

Data were assembled and analysed using R Statistical Software (version 4.1.0; R Core Team 2021). Structured data and data obtained using manual extraction methods were stored in separate Microsoft excel files. These files were merged and assembled using R. Data were assembled with each row representing a single *treatment episode*. A treatment episode was defined as a period during which an antipsychotic treatment was started and stopped, or continued until the end of the follow-up period (31st March 2021). Each patient included in the study had a minimum of one treatment episode. Some patients had more than one treatment episode.

Descriptive statistics were used to describe cohort characteristics. Means and standard deviations were reported for continuous variables and frequencies and percentages for categorical variables.

Survival analyses were used to estimate time to antipsychotic discontinuation for different antipsychotic medications and illustrated using Kaplan–Meier survival curves. The censor date was the date of antipsychotic discontinuation or 31st March 2021 for treatment episodes still active by the end of the follow-up. Cox regression analyses were used to investigate the associations of (a) antipsychotics with time to discontinuation, and (b) clinician-recorded side effects with time to discontinuation.

For aim (i), age, gender, ethnicity and prescription setting were included as covariates in a multivariable analysis. For aim (ii), the prescription setting and treatment episode number were included as covariates in a multivariable analysis. For aim (iv), the treatment episode number was included as a covariate in a multivariable analysis.

The prescription setting was defined as either community (outpatient) or hospital (inpatient). Prescription setting was included as a covariate as patients receiving care in an inpatient setting may have been more unwell and had opportunity to more timely treatment switches than patients initiated on an antipsychotic in the community whose frequency of follow-up would be lower than in a psychiatric hospital. Treatment episode number was defined as the number of treatment episodes prior to and including the current antipsychotic and included as a covariate to investigate whether antipsychotics started later in the treatment trajectory were associated with a higher or lower rate of discontinuation compared to antipsychotics started earlier in the treatment trajectory.

The study was undertaken and reported according to the REporting of studies Conducted using Observational Routinely-collected Data (RECORD) statement. A completed RECORD checklist is provided in the Supplemental Material.

## Results

### Descriptive statistics

Of 3312 patients accepted to a SLaM EIS between 1st April 2008 and 31st March 2019, 2309 patients were included in the study. A study attrition chart can be found in Supplemental Figure 1. The total study follow-up included 6830.8 person years. The mean and median follow-up times per patient were 34.2 months (SD = 28.7) and 24.2 months [IQR = 32.9, 95% confidence interval (CI) = 12.4–50.1], respectively. Data from 7013 treatment episodes were ascertained, of which 2309 (32.9%) were first treatment episodes. The mean number of treatment episodes per patient was 3.1 (range = 1–19). The mean age of patients was 26.7 years. 1492 (64.6%) were male and 817 (35.4%) were female. 1096 patients were Black (47.5%), 703 (30.4%) White, 194 (8.4%) Asian, 102 (4.4%) were of mixed ethnicity and 214 (9.3%) were ‘Other’ ethnicity. 1093 (47.3%) were diagnosed with schizophrenia, 107 (4.6%) with psychotic depression, 104 (4.5%) with bipolar disorder, 74 (3.2%) with substance-related psychosis and 18 (0.8%) with schizoaffective disorder and 913 (39.5%) with psychotic disorder not otherwise specified.

### Frequency of antipsychotic medications

Olanzapine, risperidone and aripiprazole were the most frequently prescribed oral antipsychotic medications, both across first treatment options (90%) and all treatment episodes (70%) (Table 1). Long-acting injectable antipsychotics (LAIs) were rarely prescribed as a first treatment, and clozapine was never prescribed as a first treatment. LAIs were used more for subsequent episodes, and paliperidone LAI, aripiprazole LAI and zuclopenthixol LAI were the most frequently prescribed LAIs. Across all episodes, only 1.7% of prescriptions were for clozapine.

**Table 1.** Frequency of antipsychotic treatment episodes.

Antipsychotic	Number of patients commenced on a specific antipsychotic as the first prescribed treatment (*n* = 2309) (%)	Number patients commenced on a specific antipsychotic across all treatment episodes (*n* = 7013) (%)
Olanzapine	1013 (43.9)	1860 (26.5)
Risperidone	571 (24.7)	1314 (18.7)
Aripiprazole	460 (19.9)	1638 (23.4)
Quetiapine	145 (6.3)	515 (7.3)
Amisulpride	85 (3.7)	396 (5.7)
Paliperidone 1-monthly LAI	0 (0.00)	264 (3.8)
Aripiprazole LAI	0 (0.00)	216 (3.1)
Haloperidol	18 (0.8)	162 (2.3)
Clozapine	0 (0.00)	117 (1.7)
Lurasidone	8 (0.4)	111 (1.6)
Zuclopenthixol LAI	4 (0.2)	97 (1.4)
Flupenthixol LAI	1 (0.04)	80 (1.1)
Haloperidol LAI	2 (0.1)	68 (1.0)
Risperidone LAI	0 (0.00)	39 (0.6)
Pipotiazine LAI	0 (0.00)	25 (0.4)
Paliperidone 3-monthly LAI	0 (0.00)	18 (0.3)
Flupenthixol	0 (0.00)	17 (0.2)
Olanzapine LAI	0 (0.00)	13 (0.2)
Sulpiride	0 (0.00)	13 (0.2)
Paliperidone	1 (0.04)	10 (0.1)
Sulpiride LAI	0 (0.00)	5 (0.1)
Cariprazine	0 (0.00)	5 (0.1)
Asenapine	0 (0.00)	4 (0.1)
Trifluoperazine	1 (0.04)	3 (0.04)
Penfluridol	0 (0.00)	3 (0.04)
Chlorpromazine	0 (0.00)	1 (0.01)
Fluphenazine	0 (0.00)	1 (0.01)

LAI, Long acting injectable.

### Discontinuation of the first prescribed antipsychotic medication

**Figure 1.**
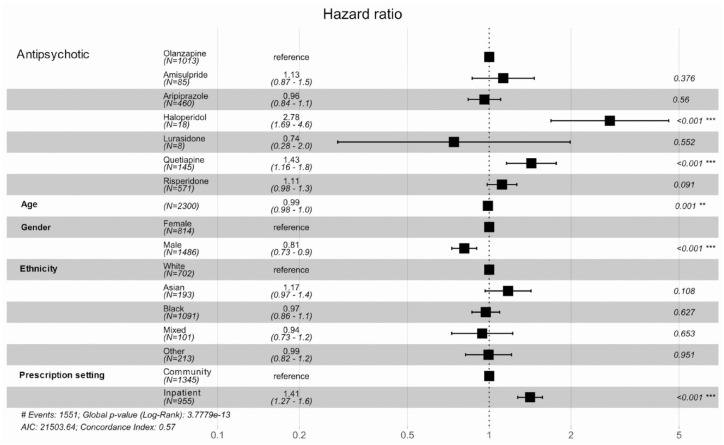
Cox regression analysis comparing the rate of discontinuation between first antipsychotic episodes.

Figure 1 and Supplemental Table 4a and b show results from a Cox regression and mean time to event analysis evaluating the association of the most frequently prescribed oral antipsychotics with time to discontinuation compared to olanzapine (the most frequently prescribed antipsychotic) for first treatment episodes. During the follow-up period, 1551 patients (67.4%) discontinued their first prescribed antipsychotic. A Kaplan–Meier survival curve is provided in Supplemental Figure 2. When prescribed as the first treatment, haloperidol [hazard ratio (HR) = 2.78, 95% CI = 1.69–4.60] and quetiapine (HR = 1.43, 95% CI = 1.16–1.80) were discontinued significantly earlier than olanzapine. Of note, for haloperidol, the mean time to discontinuation was 3.5 months (standard error: 0.97) and the median time to discontinuation was 1.2 months, whereas for olanzapine, the mean time to discontinuation was 23.5 months (standard error: 1.38) and median time to discontinuation was 9.4 months. First prescribed antipsychotics were discontinued earlier when commenced in inpatient settings than in community settings (HR = 1.41, 95% CI = 1.27–1.60). First prescribed antipsychotics were discontinued later in male patients than in female patients (HR = 0.81, 95% CI = 0.73–0.90).

### Discontinuation of antipsychotic medication across all treatment episodes

**Figure 2.**
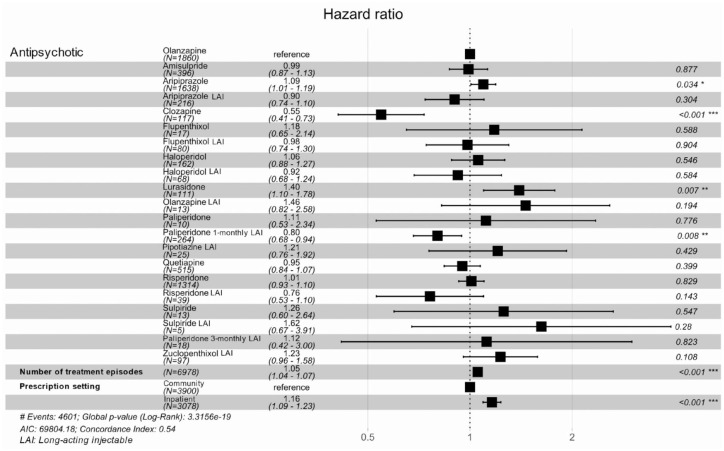
Cox regression analysis comparing the risk of discontinuation between antipsychotics prescribed at any time point.

Figure 2 and Supplemental Table 5a and b describe a Cox regression and mean time to event analysis evaluating the association of the most frequently prescribed oral antipsychotics with time to discontinuation compared to olanzapine (the most frequently prescribed antipsychotic) for all treatment episodes. Across all treatment episodes, 4601 antipsychotics (65.9%) were discontinued during the follow-up period. A Kaplan–Meier survival curve (restricted to the top five most frequently prescribed antipsychotics: amisulpride, aripiprazole, olanzapine, quetiapine and risperidone) is provided in Supplemental Figure 3. Lurasidone (HR = 1.40, 95% CI = 1.10–1.78) and aripiprazole (HR = 1.09, 95% CI = 1.01–1.19) were discontinued significantly earlier than olanzapine. Conversely, paliperidone LAI (HR = 0.80, 95% CI = 0.68–0.94) and, especially, clozapine (HR = 0.55, 95% CI = 0.41–0.73) were discontinued significantly later than olanzapine. Antipsychotics started later in patients’ treatment trajectories were discontinued earlier than those started earlier in patients’ treatment trajectories (HR = 1.05, 95% CI = 1.04–1.07). The discontinuation HR for each additional treatment episode increased by a factor of 1.05 (95% CI = 1.04–1.07). Antipsychotics prescribed in an inpatient setting were discontinued significantly earlier than antipsychotics prescribed in a community setting (HR = 1.16, 95% CI = 1.09–1.23). This may relate to more frequent medical assessment and opportunity to update treatment plans in an inpatient setting compared to a community setting. There were no other significant differences in discontinuations between olanzapine treatment episodes and other antipsychotic treatment episodes.

### Discontinuation of first prescribed antipsychotic medication by side effect incidence

**Figure 3.**
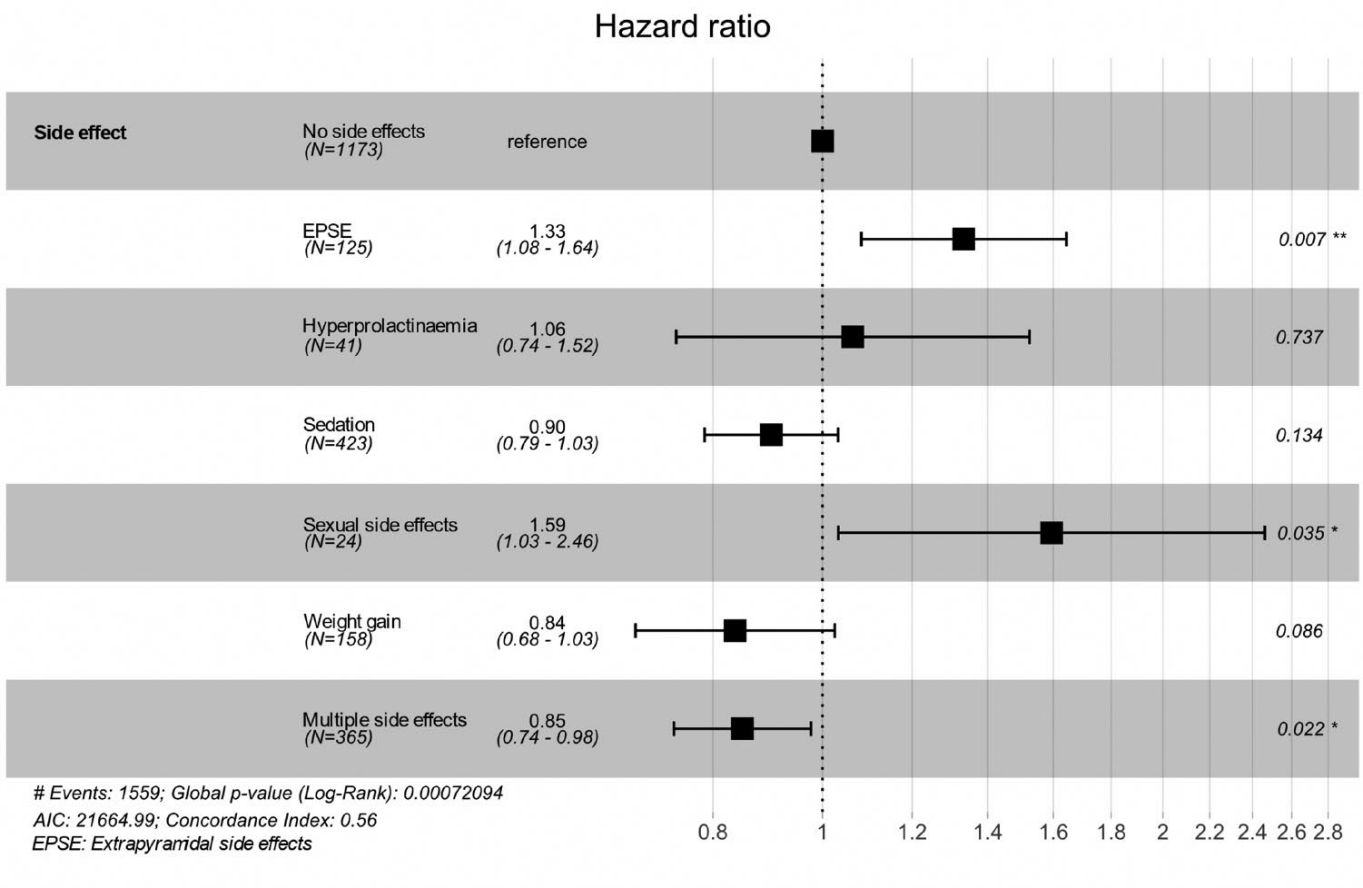
Cox regression analysis investigating the association of side effects with time to first antipsychotic discontinuation.

Figure 3 and Supplemental Table 6a and b summarize results from a Cox regression and mean time to event analysis evaluating the association of clinician-recorded side effects with time to discontinuation for first treatment episodes. A Kaplan–Meier survival curve is provided in Supplemental Figure 4. EPSEs (HR = 1.33, 95% CI = 1.08–1.64) and sexual side effects (HR = 1.59, 95% CI = 1.03–2.46) were associated with a significantly faster antipsychotic discontinuation, whereas multiple side effects (HR = 0.85, 95% CI = 0.74–0.98) were associated with a significantly later antipsychotic discontinuation than where no side effects were recorded.

### Discontinuation of antipsychotic medication by side effect data across all treatment episodes

**Figure 4.**
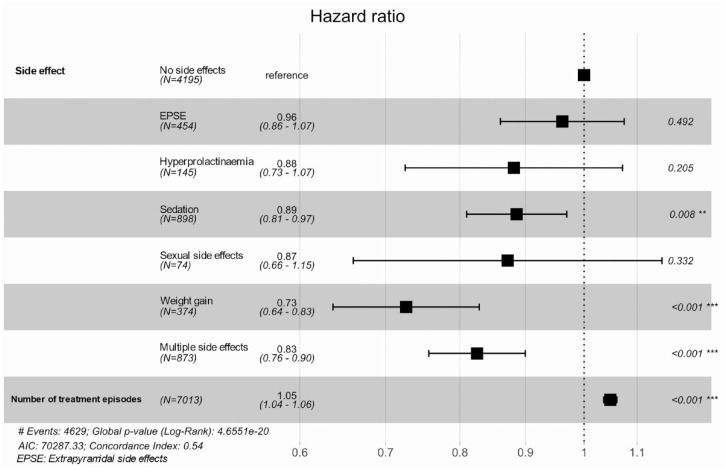
Cox regression analysis investigating the association between side effects and time to discontinuation of antipsychotic treatment at any time point in a patient’s treatment trajectory.

Figure 4 and Supplemental Table 7a and b summarize results from a Cox regression and mean time to event analysis evaluating the association of clinician-recorded side effects with time to discontinuation across all treatment episodes. A Kaplan–Meier survival curve (restricted to the top five most frequently prescribed antipsychotics) is provided in Supplemental Figure 5. Antipsychotic discontinuation was significantly delayed during treatment episodes with documented sedation (HR = 0.89, 95% CI = 0.81–0.97), weight gain (HR = 0.73, 95% CI = 0.64–0.83) or multiple side effects (HR = 0.83, 95% CI = 0.76–0.90) compared to episodes without clinician-recorded side effects.

## Discussion

This study investigated the relationship between antipsychotic discontinuation and the drug prescribed, both as first treatment and during all treatment episodes, as well as with clinician-recorded side effects. We found that there were significant differences in the time to discontinuation between different antipsychotics, both for initial and subsequent antipsychotic treatment episodes. There were also significant associations between time to discontinuation and particular adverse effects.

Although the patients were treated with a total of 27 different antipsychotic medications, three drugs (olanzapine, risperidone and aripiprazole) accounted for 90% of the initial treatments and 70% of all treatments.

### Discontinuation of first-prescribed antipsychotic medications

Patients prescribed haloperidol as the first antipsychotic medication discontinued treatment at over twice the rate of those prescribed olanzapine, with a median time to discontinuation of 1.2 months. This finding replicates the results of a meta-analysis by Zhu *et al.*,^24^ which concluded that in terms of all-cause discontinuation, haloperidol was inferior to olanzapine, aripiprazole, risperidone and quetiapine. Haloperidol is associated with a particularly high risk of motor side effects,^25^ which are poorly tolerated in people with FEP.^26^ Taken together, the findings of the present study and previous literature suggest that haloperidol should not be prescribed as a first-line treatment for patients with FEP. Quetiapine was also associated with higher rates of discontinuation than olanzapine, consistent with the findings in the CATIE^16^ and SOHO studies.^27^ This may reflect a combination of poorer efficacy and tolerability of quetiapine compared to other frequently prescribed first-line antipsychotics.^3^ Female patients discontinued treatment at a significantly greater rate than male patients consistent with findings from a previous study.^8^ This may relate to differences in pharmacodynamics of antipsychotic medications between men and women and a greater sensitivity to antipsychotic medications among female patients who may experience side effects at a lower dose than male patients.^28^

### Discontinuation of antipsychotic medications across all treatment episodes

Across all treatment episodes, aripiprazole was discontinued earlier than olanzapine. This finding contrasts with a previous result of no significant difference in discontinuation rates between antipsychotic medications in FEP patients.^11^ However, that study involved a relatively small sample, and this difference may reflect greater statistical power in the present study. A higher discontinuation rate for aripiprazole would be in line with evidence that it may be less efficacious than olanzapine.^29^ In addition, aripiprazole is more likely to be associated with akathisia and activating side effects.^24^ These side effects develop quickly, and may be less tolerated than the weight gain and metabolic side effects associated with olanzapine, which develop more slowly and insidiously. More generally, EPSEs are strongly associated with medication non-adherence,^21^ and patients with FEP are particularly sensitive to EPSEs.^26^ This finding, especially if replicated, merits consideration with respect to clinical guidelines, which may recommend aripiprazole as a first-line choice of antipsychotic for FEP.^30^

Lurasidone was also discontinued earlier than olanzapine. Like aripiprazole, lurasidone has a low propensity to cause metabolic side effects but is associated with akathisia.^31^ A further possibility is that as lurasidone is a more recently approved antipsychotic, its use may be reserved for patients with more complex or refractory illness. We attempted to adjust for factors related to clinical complexity and treatment responsiveness by including the number of treatment episodes prior to and including the current treatment episode as a covariate in multivariable Cox regression analyses. A greater number of treatment episodes may be correlated with more complex or treatment refractory illness. Nevertheless, there may be other unmeasured factors of clinical complexity that we were unable to capture from the dataset. Clozapine was associated with later discontinuation than olanzapine. Although this finding may partly reflect the relatively high levels of clinical monitoring required for patients being treated with clozapine,^32^ it could also be related to its efficacy,^29^ especially in those who have not previously benefited from antipsychotic treatment.^33–35^

Paliperidone long-acting injectable once-monthly (PP1M) was also associated with a significantly delayed discontinuation relative to olanzapine. This finding supports data from a recent meta-analysis of randomized controlled trials (RCTs), which found that LAI antipsychotics were superior to oral antipsychotics in terms of all-cause discontinuation.^36^ However, other meta-analyses of RCTs, which include selected patients signing up for such studies creating biases,^37^ have found no differences in rates of discontinuation between oral and LAI antipsychotics.^38,39^ The present study includes ten LAI antipsychotics, but only PP1M was associated with a significantly lesser discontinuation rate, suggesting that the low frequency of administration may have been a factor. However, our study is limited by small sample sizes for LAI antipsychotics and PP1M is the largest sample size of all the LAI antipsychotics in the present study. Nevertheless, multiple other database studies have reported similar findings, with PP1M being among the antipsychotics with the lowest discontinuation rate.^8,40^

### Association of clinician-recorded side effects with time to discontinuation of first-prescribed antipsychotic medications

During first-prescribed antipsychotic treatment, EPSEs were associated with greater rates of medication discontinuation compared to patients with no reported side effects. EPSEs have previously been strongly associated with medication non-adherence,^21^ and patients with FEP may be more sensitive to the effects of EPSEs than patients with chronic schizophrenia.^26^ EPSEs were one of the most frequent reported side effects in our study, second to sedation. This finding may reflect a bias in the detection of adverse effects that are often evident to clinicians and do not rely on the patient reporting them.

Despite being the least frequently reported adverse effect, sexual side effects were associated with substantially greater rates of medication discontinuation. A multi-centre, cross-sectional, naturalistic study, found that only 37% of patients experiencing antipsychotic-related sexual dysfunction spontaneously reported it.^41^ This finding has been replicated in studies of side effects associated with antidepressant medications.^42^ The strength of association may depend on whether and how carefully clinicians ask about sexual side effects, and how well these are documented. Our findings highlight the importance of eliciting the presence or absence of sexual side effects using self-reported or clinician-reported rating scales^43^ as they have been associated with reduced quality of life and functioning^44^ and may contribute to a substantially increased risk of antipsychotic discontinuation.

Recording of multiple side effects was associated with delayed antipsychotic discontinuation. There are several possible explanations to account for this. EHRs are limited by nonparticipation bias; patients who choose not to attend appointments will therefore be censored from the study earlier. Conversely, patients with multiple recorded side effects may be more adherent to antipsychotic medication, prescribed a higher dosage of medication, or more engaged with treatment leading to more detailed and more frequent clinical documentation. Patients who are more engaged and more adherent to medications may also have better psychosis symptom control. Such patients may accept a greater side effect burden and continue their medication in balance of improved symptoms.^45^

Association of clinician-recorded side effects with time to discontinuation of antipsychotic medications prescribed across all treatment episodes.

For antipsychotic treatment episodes at any point during the treatment trajectory, clinician-recorded sedation, weight gain or multiple side effects were associated with delayed antipsychotic discontinuation. This association of sedation, weight gain or multiple side effects with delayed antipsychotic discontinuation may appear counterintuitive. However, this relationship may be confounded with efficacy driving treatment persistence despite side effects, as clozapine and olanzapine are both associated with the longest treatment persistence as well as highest likelihood of sedation and weight gain.^29^ Moreover, more adherent patients and those on adequate doses may be prone to more side effects but also benefit more from the treatment.

Nevertheless, in the CATIE study, similar rates of intolerability-related discontinuation were reported for five different antipsychotics.^16^ However, the patient samples recruited to RCTs are not representative of real-world populations.^46^ For example, they may be more likely to include participants who are adherent to recommended treatment strategies and who are less likely to discontinue medication, particularly if they receive remuneration for taking part in the trial. Patients in RCTs may also have less complex presentations and may receive more clinical contact than patients in real-world samples. It is possible that side effects which occur more insidiously (e.g. weight gain) are associated with slower discontinuation rates than rapid-onset side effects (e.g. EPSEs), due to the increased time between treatment commencement and the emergence of adverse effects. Insidious side effects may take longer to be noticed by the patient or the clinician, may not become apparent in clinical trials with relatively short follow-up periods or may be tolerated by the patient in balance of symptom control by the time they have been noticed.

One surprising finding in the present study was that an absence of reported side effects was associated with a *greater* rate of discontinuation than the presence of a reported side effect or multiple side effects. Treatment episodes with no recorded side effects may represent episodes in which antipsychotics were stopped by the patient prior to clinician assessment, leading to no reported side effects but also antipsychotic discontinuation due to apparent inefficacy. Furthermore, it is likely that side effects are underreported by patients (especially those which are subjective, such as sexual side effects or akathisia) and even where they are recognised, they may not be recorded in the EHR following clinical assessment. This means that the comparison group of no recorded side effects is likely to include some patients who do actually experience side effects secondary to antipsychotic medications. In contrast, there may be a greater propensity to record the presence of side effects if there is a plan to mitigate their clinical impact or in patients receiving additional monitoring as a consequence of the choice of therapy (e.g. clozapine). Furthermore, when side effects are inquired about and documented, this may be occurring as part of a dialogue, with the clinicians showing interest in the patient’s well-being, which could increase alliance and trust as well as treatment continuation. Both of these factors could reduce the magnitude of associations of documented side effects with treatment discontinuation and may even contribute to the directionality of findings that indicate that certain side effects are associated with a reduced rate of treatment discontinuation.

### Strengths and limitations

To our knowledge, this is the largest study to investigate the associations of antipsychotic treatment and clinician-recorded side effects with treatment discontinuation in patients with FEP. The use of an EHR dataset enabled the assembly and analysis of data from a sample of 2309 patients, 7013 treatment episodes and over 6800 person years of follow-up data. Moreover, these data are representative of real-world clinical practice in a large provider of specialist mental healthcare with patients presenting to Early Intervention Services for psychosis increasing the likelihood that the population represented patients who were in the early stages of treatment for an emerging psychotic disorder. This fact increases the likelihood that inferences from the analyses are generalizable to other FEP populations.

Nevertheless, the information in EHRs is dependent on what clinicians choose to document. For example, data on clinician-observed side effects is reliant on the accurate sharing and recording of side effect information in a clinical setting. A further consideration is that patients may have been prescribed other psychotropic or non-psychotropic medications in addition to antipsychotics. However, these other medications were not routinely recorded in the mental healthcare EHR dataset analysed in the present study. For these reasons, the presence of recorded side effects may not have been related to documented antipsychotic treatment episodes, and so it was not possible to meaningfully analyse the associations of specific antipsychotic medications with clinician-recorded side effects. Due to small sample sizes of LAI antipsychotics within the FEP population, it was also not possible to make direct comparisons between LAI and oral preparations of the same antipsychotic. Moreover, owing to the nature of routinely recorded EHR data (which do not include a comprehensive recording of structured rating scales to ascertain and differentiate specific movement side effects) an analysis of individual extrapyramidal side effects was not possible.

We examined free text data to define antipsychotic treatment episodes. We defined the start date of antipsychotic medication based on the earliest recorded prescription or documentation of each antipsychotic and stop dates based on the recorded date of an antipsychotic medication being discontinued or the start date of the next antipsychotic recorded. This means that ascertained start dates may not have included antipsychotic prescriptions that were not documented in the EHR and ascertained stop dates may not have included antipsychotics discontinued (by either the patient or prescribing clinician) that were not documented in the EHR. Using this approach meant that it was also not possible to ascertain or analyse the impact of antipsychotic polypharmacy. Medication adherence was not systematically ascertained or documented in the EHR, as the data were obtained from real-world clinical practice where monitoring of adherence is not routinely conducted. Non-adherence to antipsychotic medication in FEP is variable and is greater for oral preparations than LAI preparations, with previous studies reporting figures ranging between 16%, 34% and 64%.^9,47,48^ However, these studies used data from EHRs or surveys to calculate non-adherence, so it is possible that non-adherence in the present sample was even higher. Non-adherence with medication makes it difficult to interpret associations between different antipsychotic treatments and time to discontinuation as well as between clinician-reported side effects and time to discontinuation.

Despite these limitations, the present study highlights some important findings that are relevant to the use of antipsychotic medications to treat FEP. These findings merit consideration when planning treatment with patients with FEP to maximize likelihood of treatment persistence and optimize clinical outcomes.

## Supplemental Material

sj-docx-1-tpp-10.1177_20451253231211575 – Supplemental material for Oral and long-acting injectable antipsychotic discontinuation and relationship to side effects in people with first episode psychosis: a longitudinal analysis of electronic health record data Click here for additional data file. Supplemental material, sj-docx-1-tpp-10.1177_20451253231211575 for Oral and long-acting injectable antipsychotic discontinuation and relationship to side effects in people with first episode psychosis: a longitudinal analysis of electronic health record data by Rashmi Patel, Aimee Brinn, Jessica Irving, Jaya Chaturvedi, Shanmukha Gudiseva, Christoph U. Correll, Paolo Fusar-Poli and Philip McGuire in Therapeutic Advances in Psychopharmacology

sj-docx-2-tpp-10.1177_20451253231211575 – Supplemental material for Oral and long-acting injectable antipsychotic discontinuation and relationship to side effects in people with first episode psychosis: a longitudinal analysis of electronic health record data Click here for additional data file. Supplemental material, sj-docx-2-tpp-10.1177_20451253231211575 for Oral and long-acting injectable antipsychotic discontinuation and relationship to side effects in people with first episode psychosis: a longitudinal analysis of electronic health record data by Rashmi Patel, Aimee Brinn, Jessica Irving, Jaya Chaturvedi, Shanmukha Gudiseva, Christoph U. Correll, Paolo Fusar-Poli and Philip McGuire in Therapeutic Advances in Psychopharmacology

## Declarations

**Ethics approval and consent to participate:** The CRIS data resource received ethical approval as an anonymized dataset for secondary analyses from Oxfordshire REC C (Ref: 18/SC/0372).

**Consent for publication:** Not applicable.

**Competing interests:** RP has received grant funding from Janssen, consulting fees from Holmusk, Akrivia Health, Columbia Data Analytics, Boehringer Ingelheim and Otsuka. CUC has been a consultant and/or advisor to or has received honoraria from: AbbVie, Acadia, Alkermes, Allergan, Angelini, Aristo, Boehringer-Ingelheim, Cardio Diagnostics, Cerevel, CNX Therapeutics, Compass Pathways, Darnitsa, Gedeon Richter, Hikma, Holmusk, IntraCellular Therapies, Janssen/J&J, Karuna, LB Pharma, Lundbeck, MedAvante-ProPhase, MedInCell, Merck, Mindpax, Mitsubishi Tanabe Pharma, Mylan, Neurocrine, Newron, Noven, Otsuka, Pharmabrain, PPD Biotech, Recordati, Relmada, Reviva, Rovi, Seqirus, SK Life Science, Sunovion, Sun Pharma, Supernus, Takeda, Teva and Viatris. He provided expert testimony for Janssen and Otsuka. He served on a Data Safety Monitoring Board for Lundbeck, Relmada, Reviva, Rovi, Supernus and Teva. He has received grant support from Janssen and Takeda. He received royalties from UpToDate and is also a stock option holder of Cardio Diagnostics, Mindpax, LB Pharma and Quantic. PFP has received research funds or personal fees from Lundbeck, Angelini, Menarini, Sunovion, Boehringer Ingelheim, Mindstrong and Proxymm Science. The other authors report no financial relationships with commercial interests.

**Availability of data and materials:** Not applicable.
