# Supplementary material for: Oral and long-acting injectable antipsychotic discontinuation and relationship to side effects in people with first episode psychosis: a longitudinal analysis of electronic health record data
Source: Ther Adv Psychopharmacol. 2023 Dec 15;13:20451253231211575. doi: 10.1177/20451253231211575 (PMC10725124; doi:10.1177/20451253231211575)

Supplementary Materials

Oral and long-acting injectable antipsychotic discontinuation and relationship to side effects in people with first episode psychosis: a longitudinal analysis of electronic health record data.

Rashmi Patel^1*^, Aimee Brinn^1*^, Jessica Irving^1^, Jaya Chaturvedi^1^, Shanmukha Gudiseva^2^, Christoph U. Correll^3,4,5^, Paolo Fusar-Poli^2,6,7^ and Philip McGuire^8,9^

1. Department of Psychological Medicine, Institute of Psychiatry, Psychology and Neuroscience, King's College London, London, UK

2. South London and Maudsley NHS Foundation Trust, London, UK

3. Department of Child and Adolescent Psychiatry, Psychosomatic Medicine and Psychotherapy, Charité – Universitaetsmedizin Berlin, corporate member of Freie Universitaet Berlin, Humboldt Universitaet zu Berlin, and Berlin Institute of Health, Berlin, Germany

4. Department of Psychiatry, The Zucker Hillside Hospital, Northwell Health, Glen Oaks, NY, USA

5. Department of Psychiatry and Molecular Medicine, Zucker School of Medicine at Hofstra/Northwell, Hempstead, NY, USA

6. Department of Psychosis Studies, Institute of Psychiatry, Psychology and Neuroscience, King's College London, London, UK

7. Department of Brain and Behavioural Sciences, University of Pavia, Italy

8. Department of Psychiatry, University of Oxford, Oxford, UK

9. Oxford NIHR Biomedical Centre, Oxford, UK

*Equal contribution to authorship

Correspondence to: Rashmi Patel ([rashmi.patel@kcl.ac.uk](mailto:rashmi.patel@kcl.ac.uk))

Department of Psychological Medicine, Institute of Psychiatry, Psychology and Neuroscience, King's College London, London, UK

Table of contents

Supplementary Table 1. Data extracted from structured fields within the CRIS database. 3

Supplementary Table 2. Data manually extracted from free text within patient records in the CRIS database. 4

Supplementary Table 3. List of antipsychotic search terms 5

Supplementary Table 4a. Cox regression to compare rates of discontinuation between first prescribed antipsychotics. 6

Supplementary Table 4b. Time to discontinuation between first prescribed antipsychotics. 7

Supplementary Table 5a. Cox regression to compare rates of medication discontinuation between different antipsychotic treatment episodes. 8

Supplementary Table 5b. Time to discontinuation between different antipsychotics across all treatment episodes. 9

Supplementary Table 6a. Cox regression investigating the association of side effects with time to first antipsychotic discontinuation. 10

Supplementary Table 6b. Time to discontinuation related to different side effects for first prescribed antipsychotics. 11

Supplementary Table 7a. Cox regression investigating the association between side effects and time to discontinuation of antipsychotic treatment, at any time point in a patients’ treatment trajectory. 12

Supplementary Table 7b. Time to discontinuation related to different side effects across all treatment episodes. 13

Supplementary Figure 1. Study attrition Chart 14

Supplementary Figure 2. Kaplan-Meier survival curve to illustrate time to discontinuation of first prescribed antipsychotics. 15

Supplementary Figure 3. Kaplan-Meier survival curve comparing time to discontinuation of antipsychotics prescribed at any time point, restricted to the top 5 most frequently prescribed antipsychotics 16

Supplementary Figure 4. Kaplan-Meier survival curve comparing time to discontinuation of first prescribed antipsychotic associated with clinician-recorded side effects. 17

Supplementary Figure 4. Kaplan-Meier survival curve comparing time to discontinuation of antipsychotics prescribed at any time point associated with clinician-recorded side effects. 18

## Supplementary Table 1. Data extracted from structured fields within the CRIS database.

| **Variable** | **Data type** | **Description** |
| --- | --- | --- |
| BRCID | Character | Unique patient identifier. |
| Accepted_date | Date | The date that the patient was accepted to an EIS service. |
| EIS_team | Categorical | The EIS team which accepted the patient. EIS teams were ‘Lambeth Early Onset (LEO) team’, ‘Southwark Team for Early Psychosis (STEP), ‘Lewisham Early Intervention Service’, ‘Croydon Outreach and Assertive Support Team (COAST)’. |
| Age | Continuous | The age of the patient at acceptance to EIS team. |
| Gender | Categorical | The gender assigned to the patient at acceptance to EIS team. |
| Ethnicity | Categorical | The ethnicity of the patient categorised into ‘Asian’, ‘Black’, ‘Mixed’, ‘Other’ and ‘White’. |
| Diagnosis | Categorical | The psychotic disorder diagnosis categorised into substance-related psychosis (ICD-10 F1x.5), schizophrenia and related (ICD-10 F2*, excluding F25), schizoaffective disorder (ICD-10 F25), bipolar disorder (ICD-10 F30*/F31*), psychotic depression (ICD-10 F32.3/F33.3), psychotic disorder not otherwise specified |
| Date_of_death | Date | The date of the death if the patient has died. |

## Supplementary Table 2. Data manually extracted from free text within patient records in the CRIS database.

| **Variable** | **Data type** | **Description** |
| --- | --- | --- |
| Drug_depot | Categorical | The name of the antipsychotic, suffixed by ‘0’ for oral formulations or ‘1’ for long-acting injectable formulations. |
| Start_date | Date | The date of the first prescription *or* the earliest record where the patient has been prescribed the antipsychotic. |
| Stop_date | Date | The date the prescription is ceased *or* the start date of the next antipsychotic. |
| EPSE | Binary | Coded ‘1’ if patient or clinician observes extrapyramidal side effect in the treatment episode. |
| Hyperprolactinaemia | Binary | Coded ‘1’ if evidence of hyperprolactinaemia in the treatment episode. |
| Sedation | Binary | Coded ‘1’ if patient or clinician observes sedation in the treatment episode. |
| Sexual_side_effect | Binary | Coded ‘1’ if evidence of sexual side effect in the treatment episode. |
| Weight_gain | Binary | Coded ‘1’ if patient or clinician observes weight gain in the treatment episode |

## Supplementary Table 3. List of antipsychotic search terms

abilify

aripiprazole

amisulpride

maintena

clozapine

flupentixol

flupenthixol

depixol

modecate

haloperidol

haldol

lurasidone

olanzapine

zypadhera

paliperidone

xeplion

trevicta

penfluridol

pericyazine

pimozide

piportil

promazine

quetiapine

risperidone

consta

risperdal

sulpiride

trifluoperazine

ziprasidone

zuclopenthixol

clopixol

## Supplementary Table 4a. Cox regression to compare rates of discontinuation between first prescribed antipsychotics.

|  | **Association with discontinuation (n=2,300) – Hazard Ratio (95% CI)** | | | |
| --- | --- | --- | --- | --- |
|  | **Unadjusted HR (95% CI)** | **p value** | **Adjusted HR (95% CI) †** | **p value** |
| **Antipsychotic** |  |  |  |  |
| Olanzapine (n=1,013) | *Reference* |  | *Reference* |  |
| Amisulpride (n=85) | 0.98 (0.76 to 1.30) | 0.88 | 1.13 (0.87 to 1.50) | 0.38 |
| Aripiprazole (n=460) | 0.95 (0.83 to 1.10) | 0.46 | 0.96 (0.84 to 1.10) | 0.56 |
| Haloperidol (n=18) | 2.90 (1.77 to 4.80) | <0.001*** | 2.78 (1.69 to 4.60) | <0.001*** |
| Lurasidone (n=8) | 0.67 (0.25 to 1.80) | 0.43 | 0.74 (0.28 to 2.00) | 0.55 |
| Quetiapine (n=145) | 1.29 (1.05 to 1.60) | 0.016* | 1.43 (1.16 to 1.80) | <0.001*** |
| Risperidone (n=571) | 1.07 (0.95 to 1.20) | 0.28 | 1.11 (0.98 to 1.30) | 0.091 |
| **Age (n= 2,300)** | 0.99 (0.98 to 1.00) | 0.003** |  |  |
| **Gender** |  |  |  |  |
| Female (n=814) | *Reference* |  | *Reference* |  |
| Male (n=1,486) | 0.84 (0.76 to 0.93) | <0.001*** | 0.81 (0.73 to 0.90) | <0.001*** |
| **Ethnicity** |  |  |  |  |
| White (n=702) | *Reference* |  | *Reference* |  |
| Asian (n=193) | 1.13 (0.93 to 1.40) | 0.22 | 1.17 (0.97 to 1.40) | 0.11 |
| Black (n=1,091) | 1.04 (0.92 to 1.20) | 0.53 | 0.97 (0.86 to 1.10) | 0.63 |
| Mixed (n=101) | 0.96 (0.74 to 1.20) | 0.76 | 0.94 (0.73 to 1.20) | 0.65 |
| Other (n=213) | 0.99 (0.81 to 1.20) | 0.90 | 0.99 (0.82 to 1.20) | 0.95 |
| **Prescription setting** |  |  |  |  |
| Community (n=1,343) | *Reference* |  | *Reference* |  |
| Inpatient (n=955) | 1.40 (1.20 to 1.50) | <0.001*** | 1.41 (1.27 to 1.60) | <0.001*** |
| † Multivariable analyses adjusted for age, gender, ethnicity, and prescription setting  * p < 0.05, ** p < 0.01, *** p < 0.001 | | | | |

## Supplementary Table 4b. Time to discontinuation between first prescribed antipsychotics.

| **Antipsychotic** | **Number of patients** | **Number of discontinuation events** | **Mean time to discontinuation (months)*** | **Standard error (months)** | **Median time to discontinuation (months)** | **0.95 Lower Confidence Limit** | **0.95 Upper Confidence Limit** |
| --- | --- | --- | --- | --- | --- | --- | --- |
| Olanzapine | 1013 | 686 | 23.5 | 1.38 | 9.4 | 8.06 | 10.75 |
| Amisulpride | 85 | 62 | 22.4 | 4.08 | 10.5 | 7.10 | 19.53 |
| Aripiprazole | 460 | 292 | 23.1 | 1.90 | 12.9 | 8.38 | 17.65 |
| Haloperidol | 18 | 16 | 3.5 | 0.97 | 1.2 | 0.43 | Not estimable |
| Lurasidone | 8 | 4 | 35.0 | 10.16 | 57.9 | 3.45 | Not estimable |
| Quetiapine | 145 | 104 | 22.7 | 4.28 | 4.7 | 3.45 | 8.38 |
| Risperidone | 571 | 387 | 21.5 | 1.97 | 8.8 | 6.35 | 11.84 |
| *Restricted mean with upper limit of 113 months | | | | | | | |

## Supplementary Table 5a. Cox regression to compare rates of medication discontinuation between different antipsychotic treatment episodes.

|  | **Association with discontinuation (n=6,978) – Hazard Ratio (95% CI)** | | | |
| --- | --- | --- | --- | --- |
|  | **Unadjusted HR (95% CI)** | **p value** | **Adjusted HR (95% CI) †** | **p value** |
| **Antipsychotic** |  |  |  |  |
| Olanzapine (n=1,860) | *Reference* |  | *Reference* |  |
| Amisulpride (n=396) | 1.01 (0.89 to 1.15) | 0.88 | 0.99 (0.87 to 1.13) | 0.88 |
| Aripiprazole (n=1,638) | 1.11 (1.02 to 1.21) | 0.011* | 1.09 (1.01 to 1.19) | 0.034* |
| Aripiprazole LAI (n=216) | 1.09 (0.90 to 1.33) | 0.36 | 0.90 (0.74 to 1.10) | 0.30 |
| Clozapine (n=117) | 0.71 (0.53 to 0.95) | 0.019* | 0.55 (0.41 to 0.73) | <0.001*** |
| Flupenthixol (n=17) | 1.30 (0.72 to 2.35) | 0.39 | 1.18 (0.65 to 2.14) | 0.59 |
| Flupenthixol LAI (n=80) | 1.16 (0.88 to 1.53) | 0.30 | 0.98 (0.74 to 1.30) | 0.90 |
| Haloperidol (n=162) | 1.22 (1.02 to 1.46) | 0.027* | 1.06 (0.88 to 1.27) | 0.55 |
| Haloperidol LAI (n=68) | 1.16 (0.87 to 1.56) | 0.31 | 0.92 (0.68 to 1.24) | 0.58 |
| Lurasidone (n=111) | 1.60 (1.26 to 2.03) | <0.001*** | 1.40 (1.10 to 1.78) | 0.007** |
| Olanzapine LAI (n=13) | 1.71 (0.97 to 3.03) | 0.064 | 1.46 (0.82 to 2.58) | 0.19 |
| Paliperidone (n=10) | 1.34 (0.64 to 2.82) | 0.44 | 1.11 (0.53 to 2.34) | 0.78 |
| Paliperidone 1-monthly LAI (n=264) | 0.95 (0.81 to 1.12) | 0.54 | 0.80 (0.68 to 0.94) | 0.008** |
| Pipotiazine LAI (n=25) | 1.35 (0.85 to 2.16) | 0.20 | 1.21 (0.76 to 1.92) | 0.43 |
| Quetiapine (n=515) | 0.96 (0.85 to 1.08) | 0.45 | 0.95 (0.84 to 1.07) | 0.40 |
| Risperidone (n=1,314) | 1.02 (0.94 to 1.11) | 0.65 | 1.01 (0.93 to 1.10) | 0.83 |
| Risperidone LAI (n=39) | 0.81 (0.56 to 1.17) | 0.26 | 0.76 (0.53 to 1.10) | 0.14 |
| Sulpiride (n=13) | 1.36 (0.65 to 2.85) | 0.42 | 1.26 (0.60 to 2.64) | 0.55 |
| Sulpiride LAI (n=5) | 1.77 (0.73 to 4.26) | 0.20 | 1.62 (0.67 to 3.91) | 0.28 |
| Paliperidone 3-monthly LAI (n=18) | 1.35 (0.51 to 3.61) | 0.55 | 1.12 (0.42 to 3.00) | 0.82 |
| Zuclopenthixol LAI (n=97) | 1.50 (1.17 to 1.92) | 0.001** | 1.23 (0.96 to 1.58) | 0.11 |
| **Number of treatment episodes (n=6,978)** | 1.10 (1.00 to 1.10) | <0.001** | 1.05 (1.04 to 1.07) | <0.001*** |
| **Prescription setting** |  |  |  |  |
| Community (n=3,900) | *Reference* |  | *Reference* |  |
| Inpatient (n=3,078) | 1.20 (1.10 to 1.20) | <0.001** | 1.16 (1.09 to 1.23) | <0.001*** |
| † Multivariable analyses adjusted for number of treatment episodes and prescription setting  * p < 0.05, ** p < 0.01, *** p < 0.001  N.B. This model violates the proportional hazards assumption, χ = 88.7, df = 24, p = 2.3e-09. | | | | |

## Supplementary Table 5b. Time to discontinuation between different antipsychotics across all treatment episodes.

| **Antipsychotic** | **Number of patients** | **Number of discontinuation events** | **Mean time to discontinuation (months)*** | **Standard error (months)** | **Median time to discontinuation (months)** | **0.95 Lower Confidence Limit** | **0.95 Upper Confidence Limit** |
| --- | --- | --- | --- | --- | --- | --- | --- |
| Olanzapine | 1860 | 1231 | 48.4 | 0.89 | 40.4 | 38.00 | 42.60 |
| Amisulpride | 396 | 289 | 49.3 | 1.70 | 41.6 | 38.30 | 50.50 |
| Aripiprazole | 1638 | 1028 | 45.5 | 0.89 | 40.0 | 37.30 | 42.90 |
| Aripiprazole LAI | 216 | 111 | 46.3 | 2.52 | 47.0 | 42.40 | 52.10 |
| Clozapine | 117 | 49 | 57.0 | 3.57 | 60.7 | 48.10 | 82.00 |
| Flupenthixol | 17 | 11 | 42.3 | 7.25 | 39.5 | 21.80 | Not estimable |
| Flupenthixol LAI | 80 | 52 | 44.7 | 3.95 | 40.4 | 25.10 | 54.80 |
| Haloperidol | 162 | 135 | 43.1 | 2.35 | 39.1 | 31.20 | 45.80 |
| Haloperidol LAI | 68 | 47 | 45.4 | 3.37 | 45.6 | 32.60 | 58.20 |
| Lurasidone | 111 | 72 | 36.8 | 2.03 | 36.2 | 33.70 | 46.50 |
| Olanzapine LAI | 13 | 12 | 36.4 | 6.36 | 39.5 | 17.90 | Not estimable |
| Paliperidone | 10 | 7 | 42.1 | 8.07 | 31.9 | 25.80 | Not estimable |
| Paliperidone 1-monthly LAI | 264 | 173 | 51.5 | 2.02 | 46.9 | 43.00 | 55.00 |
| Pipotiazine LAI | 25 | 18 | 35.5 | 7.81 | 23.5 | 19.20 | 73.20 |
| Quetiapine | 515 | 338 | 50.2 | 1.65 | 44.3 | 38.40 | 51.80 |
| Risperidone | 1314 | 916 | 47.7 | 1.03 | 40.3 | 37.90 | 43.60 |
| Risperidone LAI | 39 | 30 | 51.4 | 7.64 | 22.9 | 17.00 | 88.80 |
| Sulpiride | 13 | 7 | 40.3 | 7.85 | 45.3 | 19.80 | Not estimable |
| Sulpiride LAI | 5 | 5 | 35.0 | 9.31 | 47.7 | 13.70 | Not estimable |
| Paliperidone 3-monthly LAI | 18 | 4 | 29.7 | 3.46 | 25.3 | 20.00 | Not estimable |
| Zuclopenthixol LAI | 97 | 66 | 37.8 | 2.83 | 33.1 | 25.10 | 44.50 |
| *Restricted mean with upper limit of 181 months | | | | | | | |

## Supplementary Table 6a. Cox regression investigating the association of side effects with time to first antipsychotic discontinuation.

|  | **Association with discontinuation (n=2,309)** | |
| --- | --- | --- |
|  | **Unadjusted HR (95% CI)** | **p value** |
| **Side effect** |  |  |
| No side effects (n=1,173) | *Reference* |  |
| EPSE (n=125) | 1.33 (1.08 to 1.64) | 0.007** |
| Hyperprolactinaemia (n=41) | 1.06 (0.74 to 1.52) | 0.74 |
| Sedation (n=423) | 0.90 (0.79 to 1.03) | 0.13 |
| Sexual side effect (n=24) | 1.59 (1.03 to 2.46) | 0.035* |
| Weight gain (n=158) | 0.84 (0.68 to 1.03) | 0.086 |
| Multiple side effects (n=365) | 0.85 (0.74 to 0.98) | 0.022* |
| * p < 0.05, ** p < 0.01, *** p < 0.001  N.B. This model violates the proportional hazards assumption, χ = 125, df = 6, p < 2×10^-16^. | | |

## Supplementary Table 6b. Time to discontinuation related to different side effects for first prescribed antipsychotics.

| **Side effect** | **Number of patients** | **Number of discontinuation events** | **Mean time to discontinuation (months)*** | **Standard error (months)** | **Median time to discontinuation (months)** | **0.95 Lower Confidence Limit** | **0.95 Upper Confidence Limit** |
| --- | --- | --- | --- | --- | --- | --- | --- |
| No side effects | 1173 | 741 | 24.8 | 1.62 | 7.3 | 5.88 | 9.44 |
| EPSE | 125 | 100 | 14.5 | 2.27 | 3.7 | 1.61 | 6.44 |
| Hyperprolactinaemia | 41 | 31 | 15.1 | 2.63 | 6.9 | 3.19 | 22.29 |
| Sedation | 423 | 289 | 22.8 | 1.82 | 11.1 | 8.45 | 14.07 |
| Sexual side effects | 24 | 21 | 15.0 | 7.12 | 2.6 | 1.38 | 7.20 |
| Weight gain | 158 | 107 | 24.1 | 3.78 | 11.3 | 9.01 | 15.16 |
| Multiple side effects | 365 | 270 | 23.3 | 1.92 | 12.6 | 10.42 | 16.08 |
| *Restricted mean with upper limit of 113 months | | | | | | | |

## Supplementary Table 7a. Cox regression investigating the association between side effects and time to discontinuation of antipsychotic treatment, at any time point in a patients’ treatment trajectory.

|  | **Association with discontinuation (n=7,013) - Hazard Ratio (HR)** | | | |
| --- | --- | --- | --- | --- |
|  | **Unadjusted HR (95% CI)** | **p value** | **Adjusted HR (95% CI) †** | **p value** |
| **Side effect** |  |  |  |  |
| No side effects (n=4,195) | *Reference* |  | *Reference* |  |
| EPSE (n=454) | 0.96 (0.86 to 1.07) | 0.46 | 0.96 (0.86 to 1.08) | 0.49 |
| Hyperprolactinaemia (n=145) | 0.89 (0.74 to 1.09) | 0.26 | 0.88 (0.73 to 1.07) | 0.21 |
| Sedation (n=898) | 0.86 (0.78 to 0.94) | <0.001*** | 0.89 (0.81 to 0.97) | 0.008** |
| Sexual side effects (n=74) | 0.86 (0.66 to 1.14) | 0.30 | 0.87 (0.66 to 1.15) | 0.33 |
| Weight gain (n=374) | 0.71 (0.62 to 0.81) | <0.001*** | 0.73 (0.64 to 0.83) | <0.001*** |
| Multiple side effects (n=873) | 0.81 (0.74 to 0.89) | <0.001*** | 0.82 (0.76 to 0.90) | <0.001*** |
| Number of treatment episodes (n=7,013) |  | | 1.05 (1.04 to 1.06) | <0.001*** |
| †Adjusted for number of treatment episodes | | | | |
| * p < 0.05, ** p < 0.01, *** p< 0.001  N.B. This model violates the proportional hazards assumption, χ = 100.3, df =7, p < 2×10^-16^. | | | | |

## Supplementary Table 7b. Time to discontinuation related to different side effects across all treatment episodes.

| **Side effect** | **Number of patients** | **Number of discontinuation events** | **Mean time to discontinuation (months)*** | **Standard error (months)** | **Median time to discontinuation (months)** | **0.95 Lower Confidence Limit** | **0.95 Upper Confidence Limit** |
| --- | --- | --- | --- | --- | --- | --- | --- |
| No side effects | 4195 | 2656 | 45.1 | 0.56 | 39.5 | 37.90 | 41.10 |
| EPSE | 454 | 351 | 46.3 | 1.58 | 38.0 | 33.40 | 43.40 |
| Hyperprolactinaemia | 145 | 105 | 47.6 | 2.96 | 39.2 | 33.60 | 48.00 |
| Sedation | 898 | 587 | 49.8 | 1.22 | 45.2 | 41.60 | 48.50 |
| Sexual side effects | 74 | 51 | 47.7 | 4.51 | 42.0 | 27.30 | 48.60 |
| Weight gain | 374 | 244 | 56.2 | 1.90 | 52.8 | 48.10 | 58.60 |
| Multiple side effects | 873 | 635 | 51.4 | 1.20 | 42.7 | 40.30 | 47.50 |
| *Restricted mean with upper limit of 181 months | | | | | | | |

## Supplementary Figure 1. Study attrition Chart


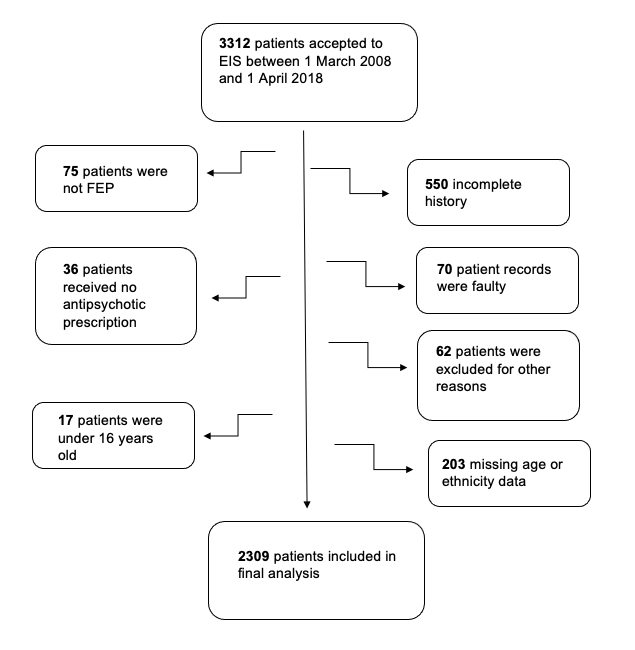


## Supplementary Figure 2. Kaplan-Meier survival curve to illustrate time to discontinuation of first prescribed antipsychotics.


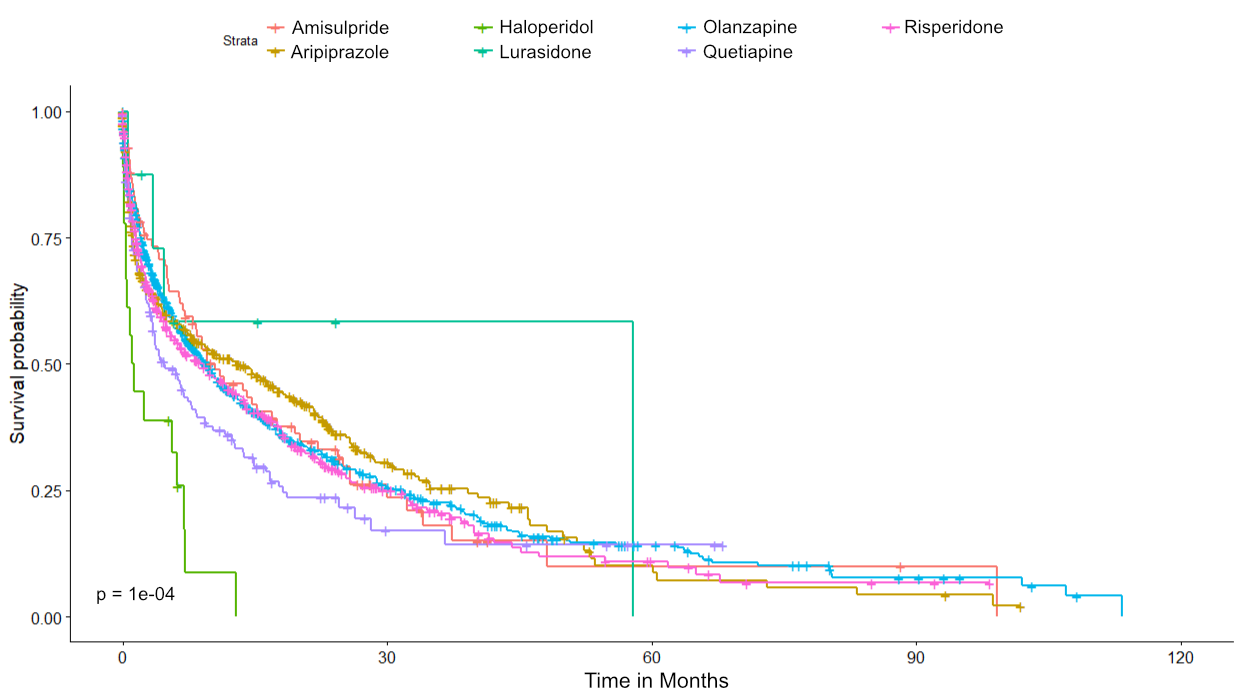


## Supplementary Figure 3. Kaplan-Meier survival curve comparing time to discontinuation of antipsychotics prescribed at any time point, restricted to the top 5 most frequently prescribed antipsychotics


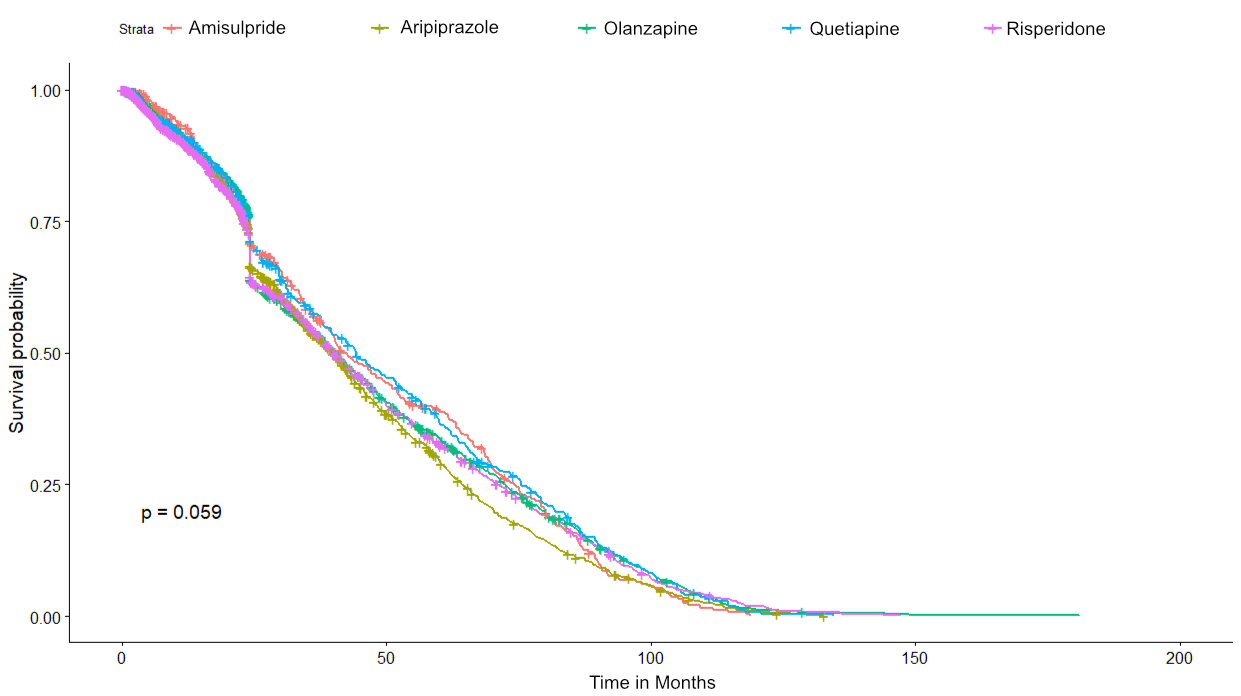


## Supplementary Figure 4. Kaplan-Meier survival curve comparing time to discontinuation of first prescribed antipsychotic associated with clinician-recorded side effects.


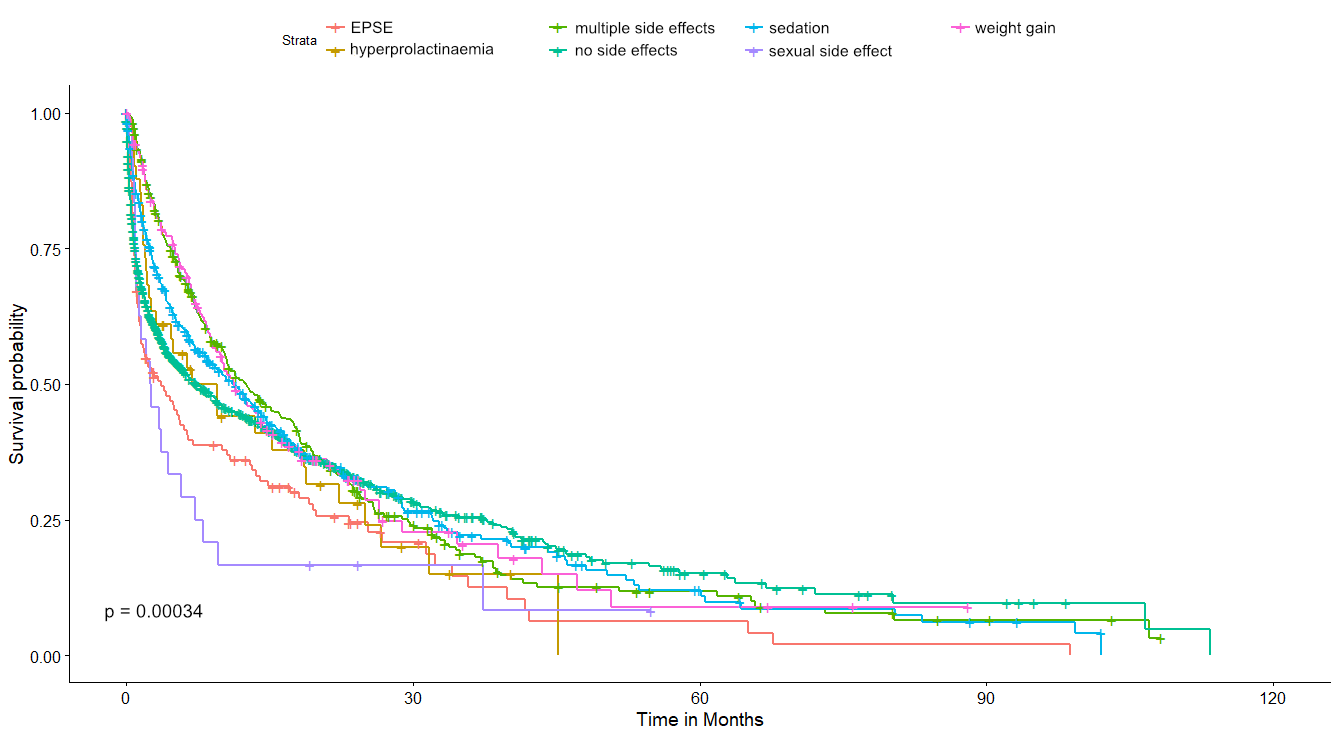


## Supplementary Figure 4. Kaplan-Meier survival curve comparing time to discontinuation of antipsychotics prescribed at any time point associated with clinician-recorded side effects.


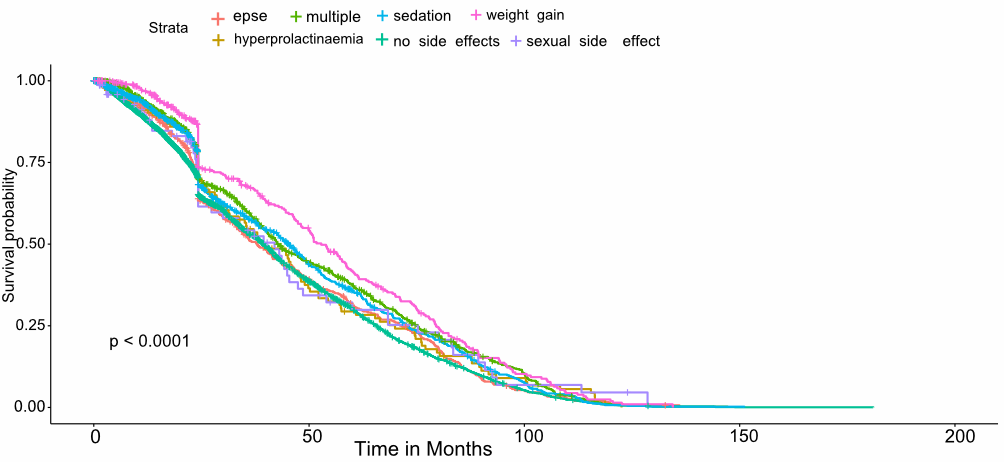

Supplement: sj-docx-1-tpp-10.1177_20451253231211575 – Supplemental material for Oral and long-acting injectable antipsychotic discontinuation and relationship to side effects in people with first episode psychosis: a longitudinal analysis of electronic health record data [file sj-docx-1-tpp-10.1177_20451253231211575.docx]
